# A systematic review of margin status in retroperitoneal liposarcomas: Does the R0 margin matter?

**DOI:** 10.3389/fonc.2022.891710

**Published:** 2022-08-11

**Authors:** Benjamin Paik, Chin Jin Seo, Joey Wee-Shan Tan, Wen Kai Darryl Juan, Khee Chee Soo, Chin-Ann Johnny Ong, Claramae Shulyn Chia, Jolene Si Min Wong

**Affiliations:** ^1^ Department of Sarcoma, Peritoneal and Rare Tumours (SPRinT), Division of Surgery and Surgical Oncology, National Cancer Centre Singapore, Singapore, Singapore; ^2^ Department of Sarcoma, Peritoneal and Rare Tumours (SPRinT), Division of Surgery and Surgical Oncology, Singapore General Hospital, Singapore, Singapore; ^3^ Lee Kong Chian School of Medicine, Nanyang Technological University, Singapore, Singapore; ^4^ Sing Health Duke-NUS Surgery Academic Clinical Program, Duke-NUS Medical School, Singapore, Singapore; ^5^ Laboratory of Applied Human GenetiCJS, Division of Medical Sciences, National Cancer Centre Singapore, Singapore, Singapore; ^6^ Sing Health Duke-NUS Oncology Academic Clinical Program, Duke-NUS Medical School, Singapore, Singapore; ^7^ Institute of Molecular and Cell Biology, ASTAR Research Entities, Singapore, Singapore

**Keywords:** liposarcoma, margin, retroperitoneal, sarcoma, R0

## Abstract

Retroperitoneal liposarcomas (RPLPSs) are a rare tumor group for which current guidelines recommend aggressive *en bloc* resection to attain microscopically negative (R0) margins. To ensure R0 margins, resection of adherent or adjacent organs is often required. However, it is still unclear if R0 margins confer any additional benefit to patients over a grossly negative but microscopically positive (R1) margin. We performed a systematic search of PubMed and Embase databases for studies including patients receiving R0 or R1 resection for RPLPS. Nine retrospective cohort studies, one prospective cohort study, and 49 case reports/case series were included. A total of 552 patients with RPLPS were evaluated: 346 underwent R0 resection and 206 underwent R1 resection. In the R0 group, 5-year overall survival (OS) ranged from 58.3% to 85.7%; local recurrence (LR) ranged from 45.5% to 52.3%. In the R1 group, 5-year OS ranged from 35% to 55.3%; LR ranged from 66.7% to 91.7%. Among cohort studies, OS, disease-free survival (DFS), LR rate, and LR-free survival (LRFS) were significantly associated with R0 resections. Assessment of case series and reports suggested that the R0 margin led to a slightly higher morbidity than that of R1. In conclusion, this review found the R0 margin to be associated with reductions in LR rates and improved OS when compared with the R1 margins, though accompanied by slight increases in morbidity. The roles of tumor histotype and perioperative chemotherapy or radiotherapy were not well-elucidated in this review.

## 1 Introduction

Retroperitoneal soft tissue sarcomas are uncommon and affect less than 0.1% of the population (1). Among them, a multitude of histological subtypes exist, with liposarcomas (LPSs) representing the most common histotype (2). Favorable survival profiles and lower propensity for distant metastases in LPS, especially in the well-differentiated (WDLPS) and low-grade dedifferentiated (DDLPS) patients (3), have generated great interest among sarcoma surgeons. For once, when tumor biology is “favorable,” the surgeon is now at the helm to possibly dictate patient outcomes *via* strategies to lower local recurrence (LR) rates.

Up-front extended resection (ER) to achieve microscopically clear (R0) margins was introduced by Gronchi et al. (4) and has been shown to significantly lower rates of LR with acceptable morbidity and mortality profile. While adopted by most of Europe and the Trans-Atlantic group (TARPSWG) (5), differing opinions continue to exist regarding the utility of such an aggressive surgical approach in the management of retroperitoneal sarcomas (RPS). Few would argue for the preservation of involved or encased organs; as such, the debate lies mainly in the *en bloc* removal of adherent or adjacent organs in which the suspicion of histological invasion is low (6, 7).

The addition of perioperative radiation therapy (RT) to the armamentarium of tools aimed at minimizing LR rates further adds complexity to the subject matter (8). It is unknown if a planned R1 (microscopically positive) margin in the context of neoadjuvant RT is of equivalence to the R0 margin. In the subset of patients with LPS, however, exploratory analysis from the STRASS trial appears to suggest a potential benefit of preoperative RT (9).

To date, data on the optimal surgical margins in retroperitoneal LPS (RPLPS) have been limited to retrospective cohort studies or case series/reports. As such, our study aims to provide a summative analysis on patients with RPLPS in an attempt to shed light on the effects of margins status, RT, chemotherapy (CT), and histotype on survival and recurrence outcomes.

## 2 Materials and methods

**Figure 1:**
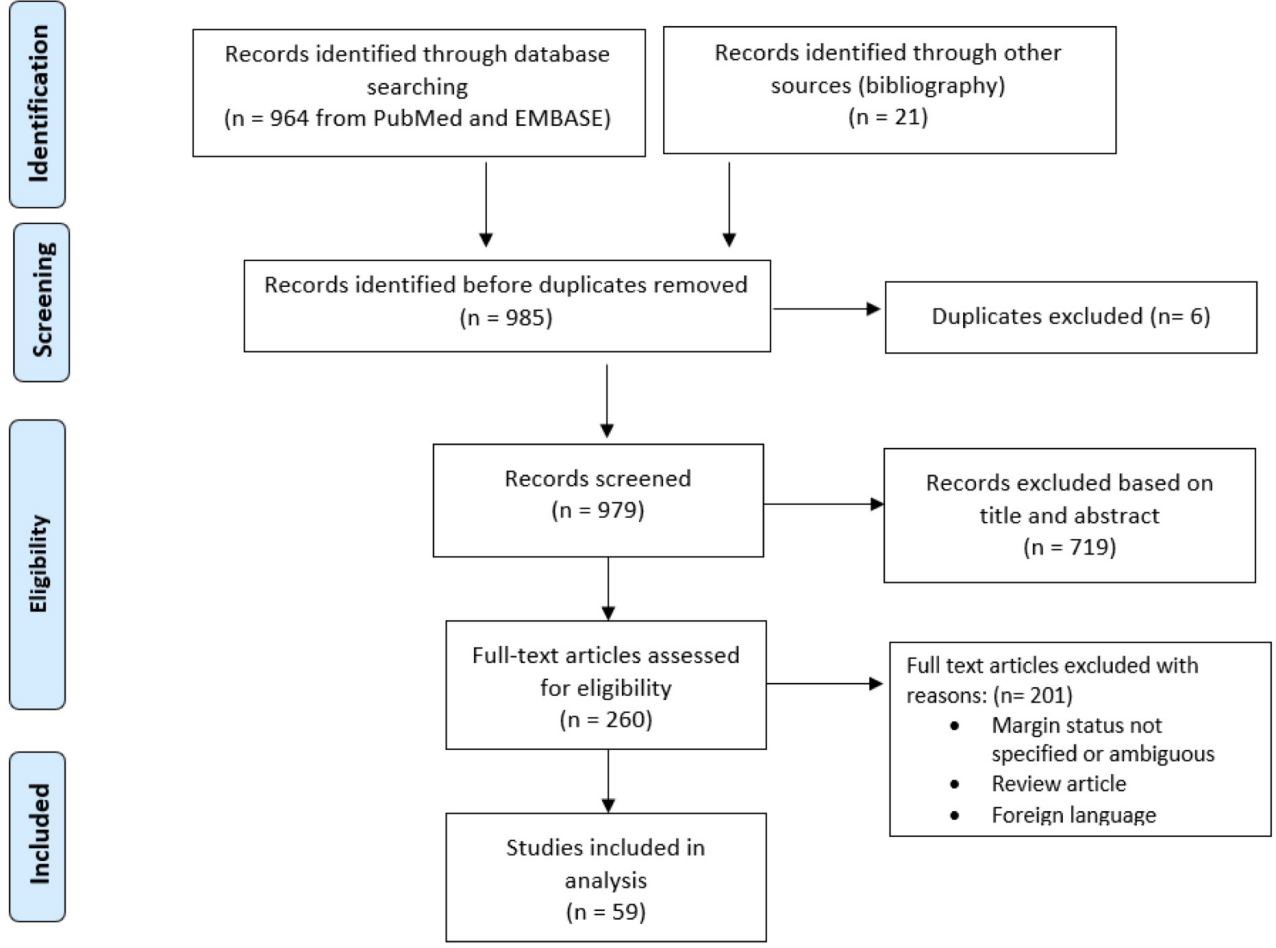
Preferred Reporting Items for Systematic Reviews and Meta-Analyses (PRISMA) flow diagram of selection of eligible studies.

A literature search of PubMed, OvidSP, Embase, and Cochrane databases was conducted for studies reporting on the surgical management of RPLPS up to March 2020. The medical search headings (MeSH) “retroperitoneal liposarcoma,” “well-differentiated liposarcoma,” “de-differentiated liposarcoma,” “R0,” “R1,” “resection,” “extended resection,” “microscopic,” and “margin” were used. Additional relevant studies were identified by screening the references cited in shortlisted articles. This study was conducted in accordance to the Preferred Reporting Items for Systematic Reviews and Meta-Analyses (PRISMA) guidelines (**Figure 1**) (10).

### 2.1 Criteria for inclusion of study

Articles were included if they 1) were original articles published in English in peer-reviewed journals; 2) included patients with RPLPS identified *via* imaging modalities such as computed tomography or magnetic resonance imaging scans; 3) included patients with biopsy-proven RPLPS; 4) unambiguously reported margin status, patient survival, and morbidity outcomes.

Articles were excluded if they 1) did not report the margin status of the resections; 2) included patients presenting with metastatic disease at initial diagnosis; 3) reported all outcomes for R0 and R1 resections collectively. Studies that presented data on RPS patients with other non-LPS histotypes were included only if data of patients with RPLPS could be extracted independently. For example, the retrospective cohort study of RPS patients from the Memorial Sloan-Kettering Cancer Center could not be included because survival and recurrence data for R1 or R0 resections were merged with other non-LPS histotypes (11). Similarly, the TAPRSWG 2020 study evaluating a large cohort of RPS patients was excluded, as outcomes for R1 and R0 resections were reported together (12).

### 2.2 Data extraction and analysis

Data were extracted using standardized forms, which recorded patient and study characteristiCJS, the radicality of resection performed (R0 or R1), histologic subtype (well-differentiated, dedifferentiated, pleomorphic, or myxoid), tumor grade (FNCLCC), postoperative morbidity and mortality, the use of neoadjuvant and/or adjuvant chemotherapy (CT) or radiotherapy (RT), number of additional organs removed and other perioperative outcomes, by two independent reviewers. Where appropriate, data that were reported for R0 and R1 collectively were extracted but were not considered in further analysis.

All studies were assessed for their level of evidence using the Oxford Centre for Evidence-Based Medicine Levels of Evidence (13). The authors elected to perform a descriptive review of the data as opposed to a meta-analysis due to the heterogeneity of the studies assessed.

### 2.3 Definitions

In accordance with residual tumor classification (R-classification) guidelines laid out by the American Joint Committee on Cancer (AJCC), an R1 resection was defined as microscopic tumor cells present at the border of the specimen, while an R0 resection was defined as the absence of tumor cells at the inked resection surface (14).

An “R+1” margin was defined as having >1 mm of normal tissue between the tumor and the inked resection margin (15).

## 3 Results

The search identified 59 relevant articles published between 1996 and 2019 (**Tables 1A**, **B**).

**Table 1A:** List of cohort studies reporting on retroperitoneal liposarcomas.

Study	Yr	Design	Study Duration	Level of Evidence	R0	R1	Outcomes reported	Description
Sanchez-Hidalgo et al. (16)	2018	Cohort study	2004-2015	2b	27	8	DFS, OS, early (<12 mo) recurrence, late (>12 mo) recurrence	Analyses influence of tumor size, stage, grade, histology, contiguous resection, BMI, age and adjuvant therapy on OS and DFS.
Nathenson et al. (17)	2018	Cohort study	2000-2013	2b	12	11	PFS, OS	Analyses influence of tumor size, stage, grade and histology and margin on OS and PFS.
Zhao et al. (18)	2015	Cohort study	2000-2007	2b	39	22	OS	Analyses the prognostic factors of postoperative outcomes. Margin status, tumor grade, ascites, postop metastasis and age were significant predictors of OS.
Sargos et al. (19)	2012	Cohort study	2007-2008	2b	4	4	RR, RFS^a^, OS^a^	Case series documenting the effect of pre-op tomotherapy on RPLPS patients.
Lee et al. (20)	2011	Cohort study	1990-2005	2b	11	10	OS, DFS, morbidity^a^, mortality	Analyses influence of tumor size, grade, histology, margin status, and age on OS and DFS.
Milone et al.(21)	2011	Cohort study	1990-2011	2b	21	6	OS, LRFS, RR	Case series documenting the overall survival and recurrence rate for R0 and R1
Singer et al. (22)	2003	Cohort study	1982-2001	2b	77	66	DSS, LRFS, DRFS	Analyses factors predicting recurrence patterns and OS. De-differentiated histology and the need for contiguous organ resection increases risk for LR; margin is prognostic for survival.
Linehan et al. (23)	2000	Cohort study	1982-1998	2b	105	54	DSS,LR, DR^a^	Analyses influence of anatomic site, margin status, tumor size and grade on LR and DSS.
Wu et al. (24)	2018	Cohort study	2005-2015	2b	0	15	DSS, LR^a^	Assesses the utility of vimentin and Ki-67 as prognostic biomarkers. R1 margins were not a prognostic factor for DSS, while gross margins were.
Rhu et al. (25)	2017	Cohort study	1998-2016	2b	0	6	OS, RFS	Reports the influence of tumor grade and histology, sex, age, margin status, adjuvant therapy on OS and DFS, and compares the postop outcomes of RPLPS and inguinoscrotal LPS

BMI, body mass index; DFS, disease-free survival; DR, distant recurrence; DRFS, distant recurrence-free survival; DSS, disease specific survival; LR, local recurrence; LRFS, local recurrence-free survival; LPS, liposarcoma; OS, overall survival; PFS, progression-free survival; RFS, recurrence-free survival; RPLPS, retroperitoneal liposarcoma; RR, recurrence rate.

^a^Outcomes were collectively reported for R0/R1.

**Table 1B:** List of case series/case reports reporting on retroperitoneal liposarcomas.

Study	Year	Margin status	Description
Fernandez-Ruiz et al. (26)	2010	4 R0 5 R1	Case series documenting the evolution of RPLPS patients
Han et al. (27)	2010	2 R0	Case series of 1) RPLPS abutting the left kidney and adrenal gland removed *via en bloc* resection with removal of those organs; 2) RPLPS encasing left kidney removed *via en bloc* resection with nephrectomy
Crisan et al. (28)	2015	2 R0	Case report of 2 patients with primary LPS of the kidney. Patient 1 is a 65 y/o man with a giant RPLPS occupying the right hemi-abdomen and compressing various abdominal organs but treated with organ-sparing complete resection of tumor. Patient 2 is a 70 y/o man with LPS in the right perirenal area displacing the right kidney and colon toward the midline, treated by *en bloc* excision together with kidney.
Daldoul et al. (29)	2017	2 R0	Case series of 1) a giant RPLPS with colonic involvement removed by hemicolectomy and nephrectomy; and 2) a giant RPLPS removed by R0 resection with nephrectomy
Yaman (30)	1996	R0	Case report of RPLPS removed *via* complete resection with nephrectomy
Susini et al. (31)	2000	R0	Case report of a 27 y/o pregnant woman with RPLPS extending from the left adnexa to the epigastric region but removed sparing the left ovary, uterus, and right adnexa
Sener et al. (32)	2004	R0	Case report of a 44 y/o woman with 2.0 cm cystic mass abutting the right kidney, treated by radical nephrectomy, adrenalectomy, and *en bloc* resection of the tumor.
Mehrotra et al. (33)	2006	R0	Case report of giant inflammatory RPLPS abutting the left kidney and pushing the IVC, aorta, and the left ureter
Calo et al. (34)	2007	R0	Case report of primary mesenteric LPS removed without intestinal resection or small bowel devascularization
Gaston et al. (35)	2007	R0	Case report of a patient whose kidney was encased by RPLPS and extended into the diaphragm, treated with *en bloc* resection with partial diaphragmatic resection
Gupta and Yadav (36)	2007	R0	Case report of a patient with RPLPS invading the kidney, treated by complete resection of the tumor with wedge resection of the renal parenchyma. (This is a case series of 2 patients but only 1 had margin specified)
Perez-Ponce et al. (37)	2008	R0	Case report of RPLPS with paravertebral involvement removed *via en bloc* resection
Yildirim et al. (38)	2008	R0	Case report of a 61 y/o man with RPLPS filling the pelvic cavity and extending to the epigastric region displacing intestines and pancreas, treated by organ-sparing complete excision.
Benseler et al. (39)	2009	R0	Case report of RPLPS removed *via en bloc* resection including the left kidney and descending colon
Goertz et al. (40)	2009	R0	Case report of RPLPS dimensions 45 cm × 35 cm × 19 cm and weighed 15.5 kg, resected *via en bloc* resection
Salemis et al. (41)	2011	R0	Case report of a 73 y/o man with RPLPS extending into the thigh
Coleblunders et al. (42)	2011	R0	Case report of recurrent RPLPS invading the thoracoabdominal wall but sparing the peritoneum, treated by an *en bloc* wide margin excision caudally down to the iliopsoas muscle and cranially up to the left adrenal.
Makni et al. (43)	2012	R0	Case report of a 60 y/o man with primary RPLPS extending from the epigastrium to the pelvic region, treated with complete but organ-sparing resection. (This paper is actually a case series, but only 1 case had sufficient data suitable for review)
Bansal et al. (44)	2013	R0	Case report of giant RPLPS with adherent ileum and ureter removed by wide excision along with ileum and ureter
Sharma et al. (45)	2013	R0	Case report of inflammatory WD RPLPS removed by wide excision
Nagy et al. (46)	2013	R0	Case report of recurrent RPLPS displacing the left kidney. Although the RPLPS recurred multiple times, only the results from the resection of the primary tumor are presented in this review.
Hoshi et al. (47)	2014	R0	Case report of RPLPS removed *via* complete resection with partial nephrectomy
Caizzone et al. (48)	2015	R0	Case report of a huge RPLPS involving the vena cava and iliac vessels removed *via en bloc* resection with nephrectomy
Kasashima et al. (49)	2015	R0	Case report of a 34 y/o woman with RPLPS after first delivery
Reznichenko (50)	2016	R0	Case report of giant RPLPS involving small bowel and mesentery removed by *en bloc* resection with small intestine
Kobayashi et al. (51)	2016	R0	Case report of recurrent RPLPS managed *via* re-resection
Machado et al. (52)	2016	R0	Case report of DDLPS of the pancreas treated with distal pancreatectomy with splenectomy and regional lymphadenectomy.
Zeng et al. (53)	2017	R0	Case report of giant RPLPS removed by *en bloc* resection
Tsiao et al. (54)	2017	R0	Case report of a patient with RPLPS who developed right sided femoral nerve neuropraxia after resection
Singal et al. (55)	2018	R0	Case report of a 55 y/o man with RPLPS occupying the abdominal cavity and displacing colon anteriorly abutting the kidney, treated by meticulous dissection to free the mass from its adhesions, hence preserving the bowel.
Ioannidis et al. (56)	2018	R0	Case report of a 55 y/o woman with giant RPLPS extending from the epigastrium into the pelvic region in contact with numerous abdominal and pelvic organs. However, the mass was excised without mention of multiorgan resection.
Agrusa et al. (57)	2019	R0	Case report of a 62 y/o woman with RPLPS removed *via en bloc* laparoscopic resection along with kidney and left adrenal gland
Argadjendra et al. (58)	2019	R0	Case report of a 30 y/0 woman with RPLPS invading the left perirenal fascia and displacing the descending colon, pancreas, and duodenum, removed *via* organ-sparing resection
Huo et al. (59)	2015	R0	Case report of a 27 y/o pregnant woman with a giant left RPLPS extending from the left kidney into the left pelvis, compressing the left kidney and ureter, treated by organ-sparing complete resection; fetus was preserved and successfully delivered subsequently.
Clar et al. (60)	2009	R0	Case report of RPLPS enclosing left kidney, removed *via* marginal resection and left nephrectomy
Hashimoto et al. (61)	2010	R0	Case report of giant RPLPS abutting the kidney and diaphragm removed *via* R0 resection
Akhoondinasab and Omranifard (62)	2011	R0	Case report of WD RPLPS abutting the aorta, kidneys, and ureters, removed *en bloc* while preserving the structures
Bhat et al. (63)	2013	R0	Case report of RPLPS encasing and displacing the left kidney anteriorly, extending cranially onto the diaphragm and inferiorly into the pelvis, treated with wide excision but organ-sparing.
Oh et al. (64)	2016	R0	Case report of RPLPS encasing the kidney and abutting the aorta removed by wide excision and organ-sparing surgery
Tanaka et al. (65)	2017	R0	Case report of huge RPLPS involving the pancreas, kidney, IVC, and aorta, removed *via en bloc* resection with resection of right kidney, duodenum, pancreatic head, IVC, and abdominal aorta
Abufkhaida and Alsalameh (66)	2019	R0	Case report of an RPLPS displacing the bowel, removed *via* gross total resection
Montenegro et al. (67)	2019	R0	Case report of an anemic 65 y/o woman with RPLPS removed *via* laparoscopic resection requiring intraoperative blood transfusion
Herzberg et al. (68)	2019	R0	Case report of a 75 y/o man presenting with anorexia with RPLPS removed *via en bloc* resection with kidney and part of diaphragm
Yang et al. (69)	2016	R0	Case report of a huge RPLPS with renal involvement removed *via en bloc* resection with nephrectomy
McCallum et al. (70)	2006	R1	Case report of a postmenopausal 47 y/o woman with RPLPS involving iliac vessels and ureter managed *via en bloc* resection with total abdominal hysterectomy and bilateral salpingo-oophorectomy
Keil et al. (71)	2008	R1	Case report of a patient with relapse of high-grade RPLPS treated with incomplete (R1) resection and adjuvant RT
Sato et al. (72)	2014	R1	Case report of RPLPS with colonic involvement treated by *en bloc* resection with right colon and right kidney.
Bruce et al. (73)	2018	R1	Case report of a patient with RPLPS vascularized by branches from external iliac artery and inferior epigastric artery, treated by *en bloc* resection removing the external iliac artery and renal fascia.
Ghose et al. (74)	2018	R1	Case report of a patient with dedifferentiated RPLPS involving inter- and infra-renal IVC, treated with *en bloc* resection with right kidney (the paper is a case series, but all other patients reported had non-LPS histology)

DDLPS, dedifferentiated liposarcoma; IVC, inferior vena cava; LPS, liposarcoma; RPLPS, retroperitoneal liposarcoma; RT, radiotherapy; WD, well-differentiated.

### 3.1 Quality of evidence

#### 3.1.1 R0-margin resection

A total of 52 studies reported on the outcomes of RPLPS patients who received R0 resection (**Tables 2A**, **3A**).

**Table 2A:** Summary of cohort studies which included patients receiving R0-margin resection.

Study	Year	No. cases	CT/RT	Post-op Morbidity	OS	DFS	LRFS	RR	Margin definition in study
Sanchez-Hidalgo et al. (16)	2018	27	Unable to extract	Clavien-Dindo **≥** III: 17.1% ^a^	Median: 93 mos (95%CI: 44.9-141) ^a^	1-yr: 81% 3-year: 22.2% Median: 22 mos	NR	Early recurrence (<12mo) = 45.5%	R+1
Nathenson et al. (17)	2018	12	Adjuvant CT: n=1 ^c^ Adjuvant/neoadjuvant RT: n= 10 ^c^	NR	2-yr: 100%	2-yr: 62%	NR	LR: 50% ^c^	R
Zhao et al. (18)	2015	39	Intraop RT: n=2 ^c^ adjuvant RT: n=7 ^c^ adjuvant CT: n=15 ^c^ adjuvant CT+RT: n=11 ^c^	0%	Median: 114 mo	NR	NR	RR: 59/61 ^b^	R
Sargos et al. (19)	2012	4	Neoadjuvant RT	Unable to extract	Unable to extract	NR	NR	0%	R
Lee et al. (20)	2011	11	NR	28.6% ^a^	3-yr: 87.5% 5-yr: 58.3%	3-yr: 62.5%	NR	52% ^a^	R+1
Milone et al. (21)	2011	21	NR	0%	5-yr; 85.7%	NR	NR	52.3%	R
Singer et al. (22)	2003	77	CT 0%	NR	3-yr: 87%	NR	3-yr: 55%	50% ^a^	R+1
Linehan et al. (23)	2000	105 (derived)	NR	NR	5-yr: 65%	NR	5-yr: 42%	25% ^d^	R

CT, chemotherapy; DFS, disease-free survival; LR, local recurrence; LRFS, local recurrence-free survival; NR, not reported; OS, overall survival; RR, recurrence rate; RT, radiotherapy; CI, confidence interval.

^a^ reported collectively for R0/R1.

^b^ reported collectively for R1/R2.

^c^ reported collectively for R0/R1/R2.

^d^ reported collectively with RPLPS of the extremity and trunk.

**Table 2B:** Summary of cohort studies that included patients receiving R1-margin resection.

Study	Year	No. cases (R1=)	CT/RT	Post-op Morbidity	OS	DFS	LRFS	RR	Margin definition in study
Sánchez-Hidalgo et al. (16)	2018	8	adjuvant CT 100% RT 100%	Clavien–Dindo **≥**III: 17.1% ^a^	Median: 93 mo (95% CI: 44.9-141) ^a^	1-yr: 25%	NR	Early recurrence rate (<12 mo) = 91.7%	R+1
Nathenson et al. (17)	2018	11	Adjuvant CT: n=1 ^c^ Adjuvant/neoadjuvant RT: n= 10 ^c^	NR	2-yr: 91%	2-yr: 44 %	NR	LR: 50% ^c^	R
Zhao et al. (18)	2015	22	Intraop RT: n=2 ^c^ adjuvant RT: n=7 ^c^ adjuvant CT: n=15 ^c^ adjuvant CT+RT: n=11 ^c^	0%	Median: 55 mo	NR	NR	RR: 59/61 ^b^	R
Sargos et al. (19)	2012	4	Neoadjuvant RT	Unable to extract	Unable to extract	NR	NR	0%	R
Lee et al. (20)	2011	10	28.6% ^a^	Reported collectively	3-yr: 88.9% 5-yr: 44.4%	3-yr: 31.7%	NR	52% ^a^	R+1
Milone et al. (21)	2003	6	NR	0%	5-yr: 33.3%	NR	NR	LR = 66.6% DM = 33.3%	R
Singer et al. (22)	2000	66	NR	NR	3-yr: 70%	3-yr probability free of distant recurrence: 87%	3-yr: 50%	50% ^a^	R+1
Linehan et al. (23)	2000	54 (derived)	NR	NR	5-yr: 35%	NR	5-yr: 47%	25% ^d^	R
Wu et al. (24)	2017	15	Collectively reported for R0/R1	NR	Median: 36.9	NR	NR	NR	R
Rhu et al. (25)	2017	6	adjuvant 66.7% (CT/RT)	66.70%	Median: 44.3mo 1-yr: 80% 5-yr: 53.3%	Median: 12.5 mo 1-yr: 66.7% 5-yr: 22.2%	NR	66.70%	R

CT, chemotherapy; DFS, disease-free survival; DM, distant metastasis; LR, local recurrence; LRFS, local recurrence-free survival; NR, not reported; OS, overall survival; RR, recurrence rate; RT, radiotherapy.

a reported collectively for R0/R1.

b reported collectively for R1/R2.

c reported collectively for R0/R1/R2.

d reported collectively with RPLPS of the extremity and trunk.

**Table 3A:** Summary of 4 case series and 40 case reports which included patients receiving R0-margin resection.

First Author	Year	Histology	Grade (FNCLCC)	CT/RT	Post-op Mortality reported	Clavien-Dindo Grade	Additional organs removed	No. of organs removed	Recurrence at last followup (Yes/No)	Follow-up duration	Patient alive at last followup?
Fernandez-Ruiz et al. (26)	2010	WDLPS	1	none given	No	NA	None	0	No	50.4mo	Yes
		WDLPS	1	none given	No	NA	None	0	No	59.1mo	Yes
		pleomorphic	2	none given	Yes (operative-related death 36 days post-op)	Grade V	left hemicolon	1	No	1 mo	No (operative-related death)
		WDLPS	1	adjuvant RT	No	NA	kidney	1	Yes	62.9 mo	Yes
Han et al. (27)	2010	WDLPS	NR	none given	No	NA	kidney, adrenal gland	2	No	1.5y	Yes
		WDLPS	NR	none given	No	NA	kidney	1	No	1.5y	Yes
Crisan et al. (28)	2015	Myxoid	2	Adjuvant CT	No	NA	None	0	Yes	18 mo	Yes
		Myxoid	2	none given	No	NA	kidney	1	Yes	3 y	Yes
Daldoul et al. (29)	2017	DDLPS	NR	none given	No	NA	kidney	1	No	12 mo	Yes
		WDLPS	NR	none given	No	NA	kidney	1	Yes	3 y	Yes
Yaman (30)	1996	WDLPS	NR	none given	No	NA	kidney	1	No	42 mo	Yes
Susini et al. (31)	2000	WDLPS	NR	none given	No	NA	fallopian tube	1	No	2y	Yes
Sener et al. (32)	2004	WDLPS	1	none given	No	NA	kidney, adrenal gland	2	No	12mo	Yes
Mehrotra et al. (33)	2006	WDLPS	NR	none given	No	NA	None	0	No	24 mo	Yes
Calo et al. (34)	2007	WDLPS	NR	Adjuvant CT	No	NA	None	0	No	33 mo	Yes
Gaston et al. (35)	2007	NR	1	none given	No	NA	left hemidiaphragm	1	No	22mo	Yes
Gupta and Yadav (36)	2007	WDLPS	NR	none given	No	NA	None	0	No	6mo	Yes
Perez-Ponce et al. (37)	2008	WDLPS	low	none given	No	NA	kidney, ureter	2	No	7y	Yes
Yildirim et al. (38)	2008	WDLPS	NR	none given	No	NA	None	0	No	3mo	Yes
Benseler et al. (39)	2009	WDLPS	1	none given	No	NA	kidney, descending colon	2	Yes	10y	Yes
Goertz et al. (40)	2009	WDLPS	NR	none given	No	NA	None	0	Yes	2y	No (died of disease)
Salemis et al. (41)	2011	WDLPS	NR	none given	No	NA	None	0	No	18mo	Yes
Coleblunders et al. (42)	2011	DDLPS	NR	none given	No	NA	diaphragm, iliopsoas muscle	2	Yes	7mo	Yes
Makni et al. (43)	2012	DDLPS	NR	none given	No	NA	None	0	Yes	1.5y	Yes
Bansal et al. (44)	2013	Mixed	NR	none given	No	NA	ileum, ureter	2	Yes	63 mo	Yes
Sharma et al. (45)	2013	WDLPS	NR	none given	No	NA	None	0	No	6 mo	Yes
Nagy et al. (46)	2013	DDLPS	low	none given	No	NA	kidney	1	Yes	8 mo	Yes
Hoshi et al. (47)	2014	WDLPS	NR	none given	No	NA	kidney	1	No	10 y	Yes
Caizzone et al. (48)	2015	Pleomorphic	NR	none given	No	NA	kidney	1	No	24 mo	Yes
Kasashima et al. (49)	2015	WDLPS	NR	none given	No	NA	kidney, adrenal gland	2	No	3 y	Yes
Reznichenko (50)	2016	Myxoid	NR	none given	No	NA	small intestine, kidney	2	Yes	7 y	Yes
Kobayashi et al. (51)	2016	DDLPS	high	none given	No	Grade III	None	0	Yes	4 y	Yes
Machado et al. (52)	2016	DDLPS	high	adjuvant CT,RT	No	NA	pancreas, spleen	2	No	5 y	Yes
Zeng et al. (53)	2017	WDLPS	1	adjuvant RT	No	Grade IV	None	0	No	8 mo	Yes
Tsiao et al. (54)	2017	NR	low	none given	No	Grade III	none	0	No	6 mo	Yes
Singal et al. (55)	2018	Myxoid	NR	none given	No	NA	none	0	Yes	16 mo	Yes
Ioannidis et al. (56)	2018	WDLPS	NR	none given	No	NA	none	0	No	4 y	Yes
Agrusa et al. (57)	2019	DDLPS	NR	none given	No	NA	kidney, adrenal gland	2	No	12 mo	Yes
Argadjendra et al. (58)	2019	WDLPS	NR	none given	No	NA	none	0	No	12 mo	Yes
Huo et al. (59)	2015	Myxoid	low	none given	No	NA	None	0	No	6 mo	Yes
Clar et al. (60)	2009	WDLPS	1	none given	No	Grade I	kidney	1	No	3y	Yes
Hashimoto et al. (61)	2010	DDLPS	2	none given	No	NA	kidney	1	No	12mo	Yes
Akhoondinasab and Omranifard (62)	2011	WDLPS	1	none given	No	Grade I	None	0	Yes	2y	Yes
Bhat et al. (63)	2013	WDLPS	NR	none given	No	NA	None	0	No	8 mo	Yes
Oh et al. (64)	2016	WDLPS	1	none given	No	NA	None	0	Yes	28 mo	Yes
Tanaka et al. (65)	2017	DDLPS	NR	none given	No	NA	kidney, head of pancreas, duodenum, IVC, abdominal aorta	5	No	16 mo	Yes
Abufkhaidaand Alsalameh (66)	2019	WDLPS	low	none given	No	NA	none	0	Yes	22 mo	Yes
Montenegro et al. (67)	2019	Pleomorphic	NR	none given	No	NA	kidney, spleen	2	No	6 mo	Yes
Herzberg et al. (68)	2019	DDLPS	low	none given	No	NA	kidney, part of diaphragm	2	No	2 y	Yes
Yang et al. (69)	2016	WDLPS	NR	adjuvant CT, RT	No	NA	None	0	No	6 mo	Yes

All time-points are taken with respect to the date of initial operation.

CT, chemotherapy; DDLPS, dedifferentiated liposarcoma; DM, distant metastasis; LR, local recurrence; MO, months; NA, not applicable NR, not reported; RT, radiotherapy; WDLPS, well-differentiated liposarcoma; Y, years.

Eight were retrospective cohort studies evaluating the relationship between margin status and recurrence and survival outcomes (16–23). R0-margin patients receiving adjuvant or neoadjuvant CT/RT were included in these studies, but data on their recurrence and survival outcomes were reported together with R1-margin patients and hence could not be extracted. Of note, three studies (16, 20, 21) adopted the stricter R+1 margin classification system that classifies margins as R0 only if the resection margins are surrounded by >1 mm of tumor-negative tissue.

Of the remaining 44 studies, 4 were case series (26–29) and 40 were case reports (30–69), both documenting the recurrence and survival outcomes of RPLPS patients receiving R0-margin resection for RPLPS.

#### 3.1.2 R1-margin resection

A total of 16 studies reported on the outcomes of RPLPS patients who received R1 resection (**Tables 2B**, **3B**). R1-margin patients receiving adjuvant or neoadjuvant CT/RT were included in these studies, but data on their recurrence and survival outcomes were reported together with R0-margin patients and hence could not be extracted.

**Table 3B:** Summary of 1 case series and 5 case reports that included patients receiving R1-margin resection.

First Author	Year	Histology	Grade (FNCLCC)	CT/RT	Postop Mortality	Clavien–Dindo Grade	Additional organs removed	No. of organs removed	Recurrence at last follow-up (Yes/No)	Follow-up duration	Patient alive at last follow-up?
Fernandez-Ruiz et al. (26)	2010	WDLPS	1	adjuvant CT	None	NA	None	0	No	31.2 mo	Yes
		myxoid	2	none given	None	NA	kidney	1	Yes	7.7 mo	No (died of disease after 7.7 mo)
		WDLPS	1	none given	None	NA	Left ovary and fallopian tube	2	Yes	35 mo	Yes
		DDLPS	2	none given	None	NA	Left kidney and adrenal gland	2	No	50.7 mo	No (death due to unknown cause at 50.7 mo)
		DDLPS	2	none given	None	NA	None	0	Yes	2.6 mo	Yes
McCallum et al. (70)	2006	DDLPS	high	none given	0%	Grade I	Uterus, cervix, both ovaries, both fallopian tubes	6	No	35 mo	Yes
Keil et al. (71)	2008	NR	3	adjuvant RT	NR	NR	None	0	Yes	1 y	Yes
Sato et al. (72)	2014	WDLPS	NR	none	None	NA	Right kidney, right colon	2	No	19 mo	Yes
Bruce et al. (73)	2018	DDLPS	high	no	None	NA	Splenic bed, external iliac vessel, renal fascia, colonic mesentery	4	No	9 mo	Yes
Ghose et al. (74)	2018	DDLPS	high	adjuvant RT	None	NA	Right kidney	1	Yes	8 mo	Yes

All time points are taken with respect to the date of initial operation.

CT, chemotherapy; DDLPS, dedifferentiated liposarcoma; DM, distant metastasis; LR, local recurrence; mo, months; NA, not applicable; NR, not reported; RT, radiotherapy; WDLPS, well-differentiated liposarcoma; y, years.

Ten were retrospective cohort studies evaluating the relationship between margin status (R0/R1) with recurrence and survival outcomes (16–25). One study (19) was a prospective cohort study examining the effect of preoperative irradiation by high-dose helical tomotherapy with a total dose of 54 Gy over 30 fractions.

Of the remaining six studies, one was a retrospective case series (26) and five were case reports (70–74), both documenting the recurrence and survival outcomes of RPLPS patients receiving R1-margin resection for RPLPS.

In total, our systematic review evaluated a total of 552 patients with RPLPS of whom 346 underwent R0-margin resection and 206 underwent R1-margin resection.

### 3.2 Outcomes of the R0 margin for retroperitoneal liposarcoma (RPLPS)

A total of 346 patients achieved R0 resections, of whom 296 patients came from cohort studies and 50 from case series/case reports.

#### 3.2.1 Cohort studies (R0)

A total of 296 patients from eight cohort studies received R0-margin resection (**Table 2A**). The rates of LR ranged from 45.5% to 52.3%. The 3-year OS and DFS ranged from 87% to 87.5% and 22.2% to 62.5%, respectively. The 5-year OS ranged from 58.3% to 85.7%. From the study by Sargos et al. (19), the recurrence rate among R0-margin patients who received preoperative RT was 0%.

Due to the heterogeneity of the data, there is little basis for comparison between studies that adopted an “R+1” margin definition (16, 20, 22) vs. studies using the “R” margin definition. For example, Lee et al. (20) who used the “R+1” definition reported a lower 5-year OS (58.3%) than Milone et al. (21) (85.7%) who used the “R” definition.

#### 3.2.2 Case series and case reports (R0)

A total of 50 patients from 44 case series/case reports received R0-margin resection. The data extracted from the case series and case reports for RPLPS patients receiving R0 resection are shown in **Table 3A** and are summarized as follows. The median follow-up duration was 22 months. The histological distribution was as follows: 58% WDLPS (n = 29), 20% DDLPS (n = 10), 10% myxoid (n = 5), 6% pleomorphic (n = 3), and 6% mixed or unreported (n = 3). Moreover, 32% (n = 16) of tumors were low-grade (G1), 12% (n = 5) were high-grade (G2/G3), and 56% (n = 28) did not report tumor grade. In addition, 54% of patients (n = 27) received multivisceral resection, of whom 28% (n = 14) of patients had one additional organ resected, 24% (n = 12) had two additional organs resected, and 2% (n = 1) had five additional organs resected. The most common organ removed was the kidney (78%, n = 21) followed by the adrenal gland (15%, n = 4), diaphragm (11%, n = 3), colon (8%, n = 2), and pancreas (8%, n = 2). Regarding adjuvant CT and RT, two patients had adjuvant CT and RT, two patients had adjuvant RT, two patients had adjuvant CT, and 44 patients had neither adjuvant CT nor RT.

The postoperative outcomes are presented as follows. The median follow-up time was 22 (range 1–120 months), and two out of 50 patients demised at the end of follow-up. Cause of the two mortalities were tumor recurrence (40) and septic shock secondary to burst abdomen (26). The recurrence rate ranged from 0% to 100%. No distant metastases were reported during the duration of follow-up. Furthermore, 12% of patients (n = 6) (26, 51, 53, 54, 60, 62) experienced postoperative complications, of which 50% were Clavien–Dindo Grade 3 and above (75).

##### 3.2.2.1 Comparing well-differentiated liposarcoma (WDLPS) vs. dedifferentiated liposarcoma (DDLPS) Patients (R0)

LR among WDLPS patients was 24% (n = 7/29) while that among DDLPS patients was 40% (n = 4/10).

##### 3.2.2.2 Comparing outcomes of adjuvant chemotherapy (CT) radiotherapy (RT) vs. no CT/RT (R0)

LR among patients who received no CT or RT, only adjuvant CT, only adjuvant RT, and adjuvant CT and RT was 31% (n = 14), 50% (n = 1), 50% (n = 1), and 0% (n = 0), respectively.

### 3.3 Outcomes of the R1 margin for retroperitoneal liposarcoma (RPLPS)

A total of 206 patients in this review received resections leading to an R1 margin, of whom 196 patients came from cohort studies and 10 from case series or case reports.

#### 3.3.1 Cohort studies (R1)

A total of 196 patients from 10 cohort studies received R1-margin resection. The rates of LR ranged from 66.7% to 91.7% (**Table 2B**). The 3-year OS ranged from 70% to 88.9%. The 5-year OS ranged from 35% to 55.3%. The 3-year LRFS was 50%, and the 5-year LRFS was 47%.

Due to the heterogeneity of the data, there is little basis for comparison between studies that adopted an “R+1” margin definition (16, 20, 22) vs. studies using the “R” margin definition.

#### 3.3.2 Case series and case reports (R1)

A total of 10 patients from six case series/case reports received R1-margin resection. The data extracted from the case series and case reports for RPLPS patients receiving R1 resection are shown in **Table 3B** and are summarized as follows. The median follow-up duration was 15.5 months. The histological distribution was as follows: 30% WDLPS (n = 3), 50% DDLPS (n = 5), 10% myxoid (n = 1), and 10% unreported (n = 1). In addition, 20% (n = 2) of tumors were low-grade (G1) and 70% (n = 7) were high-grade (G2/G3), with 10% (n = 1) unreported grade. Moreover, 70% of patients (n = 7) received multivisceral organ resection, of whom 20% (n = 2) had one additional organ resected, 30% (n = 3) had two additional organs resected, 10% (n = 1) had four additional organs resected, and 10% (n = 1) had six additional organs resected. Of the patients who received multivisceral resection, the most common organ removed was the kidney (58%, n = 4), followed by the ovary (29%, n = 2). Regarding adjuvant CT/RT, seven patients had neither CT nor RT, one patient had adjuvant CT, and two patients had adjuvant RT.

At a median follow-up of 15.5 months (range 2.6–50.7), two out of 50 patients had demised (26). Only one patient (70) experienced minor Clavien–Dindo Grade 1 postoperative complications.

##### 3.3.2.1 Comparing well-differentiated liposarcoma (WDLPS) vs. dedifferentiated liposarcoma (DDLPS) patients (R1)

LR among WDLPS patients was 33% (n = 1/3) while that among DDLPS patients was 40% (n = 2/5).

##### 3.3.2.2 Comparing outcomes of adjuvant chemotherapy (CT)/radiotherapy (RT) vs. no CT/RT (R1)

LR among patients who received neither CT nor RT was 43% (three out of seven patients), LR among patients who received only CT was 0% (zero out of one patient), and LR among patients who received only RT was 100% (two out of two patients).

### 3.4 Outcomes of patients who received neoadjuvant or adjuvant radiotherapy (RT) chemotherapy (CT)

In the cohort studies, survival and recurrence outcomes of patients receiving neoadjuvant or adjuvant CT/RT were reported collectively as R0/R1 and could not be extracted independently for aggregation across studies. However, three retrospective cohort studies individually reported on the effects of neoadjuvant or adjuvant CT/RT upon univariate or multivariate analysis, with differing results. Sánchez-Hidalgo et al. (16) reported that administering adjuvant CT or RT to patients with dedifferentiated tumor histology neither improved DFS/OS nor reduced LR rates. Similarly, Nathenson et al. (17) reported that none of adjuvant CT, neoadjuvant RT, or adjuvant RT had a significant influence on OS and PFS, regardless of tumor histology and grade. Zhao et al. (18) reported a lower median survival for patients receiving adjuvant therapy (intraoperative/postoperative RT or CT) than those who did not undergo adjuvant therapy (p = 0.03) but acknowledged selection bias due to adjuvant therapy being arranged only for patients with high-grade tumors.

## 4 Discussion

RPS accounts for 15% of all soft tissue sarcomas and represents a rare class of tumors occurring in approximately 5 per 100,000 people in Europe (76). To date, the impact of microscopic margin status (R0 *vs*. R1 margin) has never been validated in RPS. While few would defend the preservation of involved or encased organs, much of the debate lies in whether an *en bloc* approach to remove all adjacent or adherent organs should override intraoperative assessment of suspected histopathologic organ invasion (HOI). To further complicate the matter, it has been shown that up to 26% of patients in whom there was no suspicion of organ involvement actually demonstrate pathologically identified HOI; this underscores the need for a more aggressive and extended resection regardless of intraoperative assessment (7). Hence, while groups like the TARPSWG (77) and EORTC-STBSG (78) recommend *en bloc* resection to maximize the chances of achieving an R0 margin, so far, there is limited evidence to conclude if the elusive R0 margin even makes a difference to patient outcomes. As such, the role of the R0-margin status is controversial in RPS.

The results of our systematic review provide some clarity on this matter. As shown in **Tables 2A**, **B**, although the numerical values for OS and DFS vary considerably between cohort studies, the R0 margin demonstrated benefits over the R1 margin with regard to these outcomes in most individual studies. For OS, the R0 margin was prognostic for increased OS in the studies by Nathenson et al. (17), Zhao et al. (18), Milone et al. (21), Singer et al. (22), and Linehan et al. (23), while studies by Sánchez-Hidalgo et al. (16) and Lee et al. (20) did not find a statistically significant correlation between the R0/R1 margin and OS. For DFS, the R0 margin was prognostic for increased DFS in studies by Sánchez-Hidalgo et al. (16) and Nathenson et al. (17), but the study by Lee et al. (20) did not find a statistically significant correlation between the R0/R1 margin and DFS. Among the case series and case reports included in our review, the follow-up duration varied tremendously and follow-up data were limited, hence preventing any formal assessment of the benefits of the R0 margin on survival outcomes.

Additionally, while different studies adopted the R+1 classification system that requires at least 1 mm of healthy tissue around the tumor margin to qualify as R0 (in essence, an R0+1 margin), there was no obvious superiority over the standard R0 margin.

One of the biggest arguments for aggressive surgical approaches, such as frontline extended resection, is the reduction in the LR rate and hence an increase in local control. Gronchi et al. (79) showed that the 5-year LR rate was lower at 28% with the frontline extended approach compared to 48% with standard less aggressive approaches. The French Sarcoma Group (80) also cited a 3.29-fold reduced LR rate for an aggressive extended approach compared to patients who underwent simple complete resection.

The studies included in our analysis showed that the R0 margin led to a lower LR rate compared to that of the R1 margin. The LR rate for the R0 margin ranged from 45.5% to 52.3%, lower than the LR rate of the R1 margin that ranged from 66.7% to 91.7%. In particular, Sánchez-Hidalgo et al. (16) found that the R1 margin was strongly correlated with early recurrence (<12 months) on univariate analysis, and in the series by Milone et al. (21), the R0-margin LR rate was lower than the R1-margin LR rate, although no statistical significance analysis was done to reinforce these findings. The limited data for LRFS appear to corroborate the above findings. Only the studies by Singer et al. (22) and Linehan et al. (23) presented LRFS data for the R0 and R1 margin separately for comparison between R0 and R1 to be done. While Singer et al. (22) reported that the R0 margin led to longer LRFS and longer distant recurrence-free survival, the benefit over the R1 margin was not statistically significant. On the other hand, Linehan et al. (23) reported that the R1 margin paradoxically led to a longer LRFS (albeit not statistically significant).

From our analysis, there were hardly any extractable data from the cohort studies concerning survival and LR data stratified by RPLPS subtypes (WDLPS/DDLPS), although the case series and reports suggest that the R0 margin benefits LR in WDLPS patients (R0, 24%; R1, 33%) but offers no additional benefit in DDLPS patients (R0, 40%; R1, 40%). At the same time, while a more aggressive multivisceral resection would increase the chance of attaining R0 margins (77, 78), the final margin status attained potentially also depends on underlying tumor biology because more dedifferentiated RPLPS tends to be more locally invasive (6) and hence has a higher inherent tendency to invade the tumor capsule to increase the chance of margins being positive on final histopathology. It is therefore possible that despite a multivisceral resection, the margin status may end up as R1. In our dataset, out of the R0 patients, majority were WDLPS histotype, whereas of the R1 patients, majority were DDLPS histotype. Yet, the more common margin status attained in each of the WDLPS and DDLPS was still R0, suggesting that R1-margin cases are grossly underrepresented in the available literature. Hence, it is challenging to conclude regarding the extent that tumor biology and extent of resection contribute to the margin status attained just based on these limited data from case series and reports. The patients with pleomorphic and myxoid RPLPS were too few to be adequately represented, and no further analysis on their outcomes was done.

That being said, proponents of aggressive resection argue that it offers the best chances of local control that in turn drives oncologic outcomes in WDLPS and <G2 DDLPS. However, aggressive resection does not offer further benefit in high-grade DDLPS patients in whom distant metastases are the main driver of outcomes (3).

Existing large-scale studies on RPS in general are not unanimous on whether aggressive resections increase morbidity and mortality. While studies by Gronchi et al. (79) argue that aggressive resections do not increase morbidity and mortality, this is refuted by groups such as the TARPSWG (81) that argues that the removal of major organs when resecting aggressively puts patients at 1.5 times greater risk of morbidity.

In our analysis, postoperative morbidity/mortality data could only be extracted from case series and case reports; where it was extractable from cohort studies, the morbidity rate for R0 and R1 was equal (**Tables 2A**, **B**). Among the case series and reports, although there were more incidences of morbidity among R0 than R1 patients, the percentage morbidity in both patient groups was roughly equal (R0 = 12% *vs*. R1 = 10%) due to the different total numbers of patients. It is however valid to say that R0 has slightly higher morbidity as evidenced by the presence of Clavien–Dindo Grade 3, 4, and 5 complications. Postoperative mortality was low in both R0 and R1 patients, with there being only one case of mortality in R0 and none among R1 patients. On the whole, our analysis suggests that postoperative morbidity and mortality are only slightly higher for the R0 margin than those for the R1 margin in the context of RPLPS.

While the precise role of each of CT and RT in survival and LR outcomes in RPLPS is not well-established due to most studies being conducted on RPS in general, it has been reported elsewhere that standard chemotherapy has a marginal role in WDLPS due to the very low mitotic rate (82), and its use is therefore limited to metastatic and recurrent tumors (83). Furthermore, within the retroperitoneal space, the presence of radiosensitive organs, such as the pancreas, and kidney, in close proximity to the primary tumor limits the effectiveness and delivery of radiotherapy (be it neoadjuvant or adjuvant) (84).

Among the studies included in our review, analysis in studies performed by Sánchez-Hidalgo et al. (16), Nathenson et al. (17), and Zhao et al. (18) failed to find any statistically significant influence of CT/RT on survival and LR outcomes. As these are retrospective studies, there is expected to be some selection bias, since CT/RT would be offered more to patients with high-grade tumors or inherently aggressive tumor biology. Furthermore, the regimen of CT and RT was not standardized among the cohort studies and, in some instances, not specified at all. The limited follow-up data from case series and reports do not show any improvement of CT/RT to survival and LR in both R0 and R1 patients nor is there any definitive proof to address the question of whether R1 with CT/RT is of equivalence to the R0 margin.

The findings of our systematic review support and allude to the latest general consensus management guidelines for RPS published by the TARPSWG in 2021 (85). Our review showed that the R0-margin resection for RPLPS increased OS and reduced LR. Indeed, the TARPSWG recommends an extended approach to resect adherent organs regardless of expected microscopic infiltration, with removal of all ipsilateral retroperitoneal fat, especially for well-differentiated histotypes that are harder to distinguish from normal adipose tissue. For this reason, obtaining intraoperative frozen sections will not add further value to guide the extent of resection.

Our review showed that WDLPS histotypes could potentially stand to benefit more from the R0 resection than DDLPS in terms of LR. While the TARPSWG suggests that the same aggressive strategy be used for both WDLPS and DDLPS, it acknowledges that more data are required to guide operative strategies for DDLPS (especially the high-grade type); data from the ongoing STRASS2 trial will shed further light on this matter.

While the studies included in our review seems to suggest that perioperative CT/RT has no significant effect on survival and LR, the TARPSWG recommends preoperative CT to downsize the primary tumor in order to facilitate grossly complete resection. Preoperative RT should be considered only for WDLPS and low-grade DDLPS that have high risks of LR, whereas high-grade DDLPS does not benefit from preoperative RT. There is still no proven benefit of postoperative CT or RT after grossly complete resection.

### 4.1 Limitations of the analysis

Our review highlighted that the majority of available studies on this topic are retrospective in nature. Outcome data for R0 were not always reported separately from those of R1, and if it was reported separately, there was also heterogeneity in the patient populations included under the R0 and R1 groups, and each study had varying proportions of WDLPS and DDLPS patients. The heterogeneity of the data limited the authors’ ability to perform a formal meta-analysis; as such, the authors elected to perform a systematic review of the available evidence.

Inconsistencies in the definitions of margin status among the cohort studies also limited the extent to which the results could be analyzed. For example, in the case of resections that had less than 1 mm of healthy tissue around the margin, this would be classified in papers adopting the “R+1” system as R1 but classified in papers adopting the “R” system as R0. Among the case reports and case series, some of the papers used did not categorically specify if the margins were R0 or R1 but described resections as “margin-positive” or “margin-negative.”

Although the numbers of R0 and R1 patients from cohort studies are fairly equal, there were much more case series and case reports of R0 patients than those of R1 patients, possibly stemming from publication bias. As such, data for short case series and case reports were simply presented in a descriptive manner. Therefore, it is difficult to make definitive conclusions regarding the effect of CT/RT, tumor histotype, or extent of resection on survival or recurrence outcomes.

## 5 Conclusion

Among the publications included in our systematic review, there was unanimity that the R0 margin delivered statistically significant improvements to OS, and there was fairly strong evidence that the R0 margin led to increased DFS. Data heterogeneity and collective reporting of R0 and R1 outcomes prevented a direct comparison of the differences in LRFS and RR, but the evidence points toward decreased RR from R0-margin resection. A modest amount of evidence points to a roughly equal postoperative morbidity rate between R0 and R1 resection.

To date, there have been no systematic reviews on the impact of the R0 margin in the treatment of RPLPS or even RPS for that matter. On the whole, our review suggests that the R0 margin benefits survival and LR in RPLPS patients without compromising postoperative morbidity. The role of other factors such as tumor biology and the role of CT/RT, while important, have yet to be delineated. At this juncture, our review emphasizes the need for larger-scale multicenter cohort studies assessing the effect of histotype, CT/RT, and extent of resection on survival and recurrence outcomes.

## Data availability statement

The original contributions presented in the study are included in the article/supplementary material. Further inquiries can be directed to the corresponding author.

## Author contributions

Conceptualization: JSMW. Data curation: BP, JSMW. Formal analysis: BP, CJS, JW-ST, WKDJ, JSMW. Funding acquisition: JSMW. Investigation: BP, CJS, JW-ST, WKDJ, JSMW. Methodology: JSMW. Project administration: CC, CO. Resources: KCS, CC, C-AJO, JSMW. Supervision: KCS, C-AJO, CC, JSMW. Validation: BP, CS JW-ST, WKDJ, JSMW. Visualization: BP, CS. Roles/Writing - original draft: BP, JSMW. Writing - review and editing: BP, CJS, JW-ST, WKDJ, KCS, CC, C-AJO, JSMW. All authors contributed to the article and approved the submitted version.

## Funding

This study is supported by the NCCS Cancer Fund (Research) and SingHealth Duke-NUS Academic Medicine Centre, facilitated by Joint Office of Academic Medicine (JOAM). C-AJO is supported by the National Research Council Clinician Scientist-Individual Research Grant (CIRG21jun-0038). All the funding sources had no role in the study design, data interpretation or writing of the manuscript.

## Conflict of interest

The authors declare that the research was conducted in the absence of any commercial or financial relationships that could be construed as a potential conflict of interest.

## Publisher’s note

All claims expressed in this article are solely those of the authors and do not necessarily represent those of their affiliated organizations, or those of the publisher, the editors and the reviewers. Any product that may be evaluated in this article, or claim that may be made by its manufacturer, is not guaranteed or endorsed by the publisher.
